# An integrated impedance biosensor platform for detection of pathogens in poultry products

**DOI:** 10.1038/s41598-018-33972-0

**Published:** 2018-10-31

**Authors:** Jiayu Liu, Ibrahem Jasim, Amjed Abdullah, Zhenyu Shen, Lu Zhao, Majed El-Dweik, Shuping Zhang, Mahmoud Almasri

**Affiliations:** 1 University of Missouri – Columbia, Electrical and Computer Engineering, Columbia, 65211 USA; 2 University of Missouri – Columbia, School of Veterinary Medicine, Columbia, 65211 USA; 3 Lincoln University, Department of Life and Physical Sciences, Jefferson City, 65101 USA

## Abstract

This paper presents an impedance-based biosensor for rapid and simultaneous detection of *Salmonella* serotypes B, D, and E with very low concentration. The biosensor consists of a focusing region, and three detection regions. The cells focusing was achieved using a ramp down electroplated vertical electrode pair along with tilted thin film finger pairs that generate p-DEP forces to focus and concentrate the bacterial cells into the center of the microchannel, and direct them toward the detection region. The detection regions consist of three interdigitated electrode arrays (IDEA), each with 20 pairs of finger coated with a mixture of anti-*Salmonella* antibody and crosslinker to enhance the adhesion to IDEA. The impedance changes as the target *Salmonella* binds to the antibody. The biosensor has showed excellent performance as proven by the detection of a single *Salmonella* serotype B, and simultaneous detection of two *Salmonella* serotypes B and D with a limit of detection (LOD) of 8 Cells/ml in ready-to-eat turkey samples, the addition of focusing capability improved the measured signal by a factor of between 4–4.5, the total detection time of 45 minutes, selectivity of the sensor on different types of bacterial cells, and the ability to distinguish between dead and live cells.

## Introduction

The Center for Disease Control and Prevention (CDC) estimates that around 48 million people in America get sick, 128,000 are hospitalized, and 3,000 die of foodborne diseases for each year^1^. *Salmonella* is ranked as the number one in the five pathogens that contribute to domestic foodborne illnesses resulting in hospitalization and death^2^. In 2013, the Economic Research Service (ERS) from USDA has reported that the annual cost due to infection of *Salmonella* in food source is estimably 3.6 billion in US dollar, while the aggregate cost due to food recall is 77 billion annually^3^. Among all the foodborne pathogens, *Salmonella* typhimurium is the second most common serotype of *Salmonella* found in humans^4^. So a method that can provide rapid, selective and accurate detection of *Salmonella* in food products is in urgent need for a better food safety.

Currently, microbiological culture and colony counting as a traditional method remains to be the most commonly used technique for food industry^5^. This method relies on enrichment and selective cultures and subsequent colony counting^6,7^. It is the official food screening procedure established by FDA for pathogens detection of clinical and food products^8^. In addition, it requires 2−5 days by trained professionals for a definitive result, which makes this method time consuming, labor intensive and costly^9^. Nucleic acid based assays such as PCR, qPCR^10^, mPCR^11^ are well established techniques for rapid pathogens detection in food products with high specificity, sensitivity and low detection limit^12^. It is popular among the food industry because it reduced the testing time to 24 hours^13^. If the processing plant/company does not have its own lab, additional time is needed to transport samples to a lab that can perform PCR^14^. PCR cannot distinguish between live and dead bacteria and false positives may occur^15,16^. BioRad has implemented a DNA cleaning step to get rid of residual dead cells DNA which may reduce the rate of false positive results^17^. Immunological methods such like enzyme-linked immunosorbent assay (ELISA) is based on specific binding of antibody to antigen^18^. ELISA and its relevant techniques including IMS-ELISA, ELISA-PCR are based on antibody-antigens binding process. The detection step is rapid but is done after an enrichment culture, e.g., the commercially available Solus Scientific Testing Solutions can detect *Salmonella* in 36 hrs, respectively^19^. The long test turnaround time not only cuts into a product’s short shelf-life but also increases the product cost due to the need for storage space and labor needed to transport the products in and out of storage. Alternatively, if food products are released before testing is completed, the company risks releasing product that may cause a foodborne illness or outbreaks of foodborne diseases, economic losses from medical costs associated with foodborne illnesses and product recalls, and risks damage to the company’s brand or even survival. Therefore, there is an urgent need for testing device/methods that could offer a rapid, accurate detection of foodborne pathogens.

Recent development in biosensor has focused on key issues such as rapid detection, limit of detection, feasibility of operation and low cost. The biosensors are mainly based on the immunoassay principle and thus grouped into three major categories: (1) Electrochemical sensors including: (a) Amperometric biosensor. With combination of Immunomagnetic beads and amperometric biosensor, *Salmonella* were detected using screen – printed carbon electrode with the detection limit of 89 CFU/ml^20^. (b) Potentiometric biosensor. Shaibani *et al*. has described a portable nanofiber-light addressable potentiometric sensor than can detect *E. coli* low to 100 CFU/ml with only 1 hour^21^. (c) Impedimetric sensors. For example, a glassy carbon electrode modified with graphene oxide and carbon nanotubes has demonstrated a LOD of 25 CFU/ml^22^. And Wan *et al*., describe a signal-off impedimetric immune-biosensor using AuNP to detect *E. coli* with LOD of 100 CFU/ml^23^. (2) Optical based biosensor. This includes: (a) surface plasmon resonance (SPR). A SPR biosensor based on ultra-low fouling and functionalizable poly brushes has successfully detected *E. coli* and *Salmonella* in cucumber and hamburger for 57 CFU/ml and 17 CFU/ml, and 7.4 × 10^3^ CFU/ml and 11.7 × 10^3^ CFU/ml, respectively^24^. (b) Surface enhanced plasmon resonance (SERS). For example, with the use of magnetic gold nanoparticles achieved LOD for *S.aureus* and *Salmonella* of 35 CFU/ml and 15 CFU/ml, respectively^25^. (c) Colorimetric. For example, Suaifan *et al*. has designed an optical colorimetric biosensor for detection of *Staphylococcus aureus* and achieved 7 CFU/ml in pure culture and 40 CFU/ml in food culture as LOD^26^. (3) Mass based biosensor. This includes (a) acoustic wave-based biosensors. For example, a micro-nano-bio acoustic system for *Salmonella* at 2 cells/µl^27^. (b) quartz crystal microbalance (QCM) method. For example, a QCM-based platform using ssDNA aptamers has achieved detection of 10^3^ CFU/ml of *Salmonella* within only 1 hour^28^. (c) Cantilever and based biosensor. For example, a dynamic-mode cantilever biosensor without surface functionalization has detected *E. coli* at 100 Cells/ml^29^. Although many of these methods have provided promising research results, none of them have been commercialized yet. This might be due to the limit of detection, the use of microbead/nanoparticles coated antibody may have complicated the detection process. The USDA/FDA set a zero-tolerance requirement for ready to eat (RTE) products. For example, in RTE poultry products, the testing method must meet the AOAC standard for certification, i.e.,1 cell/325 gr of product.

Impedimetric based biosensing technique, as one kind of the electrochemical biosensor, has proven to be a promising technique for detection of foodborne pathogens in terms of its detection speed, accuracy, and sensitivity^30^. The impedance of the electrodes will be changed by capturing bacterial cells on electrodes.

In this study, an impedance-based biosensor for simultaneous detection of multiple *Salmonella* serotypes has been designed, fabricated, characterized, and validated using ready to eat Turkey (RTE) samples. Detection of *S. Typhimurium* with various concentrations, with and without cross-linker, were utilized using RTE Turkey samples and pure culture samples. The device’s selectivity, differentiation between dead and live cells were also validated. Initially, the surface of the detection electrodes were immobilized with bioreceptors (in our case antibodies), thus, the labeling procedure was eliminated. Impedance change were achieved after direct capture of target bacteria by specific antibody on surface of the electrode^31^. An impedance analyzer was used to monitor the impedance changes.

Dielectrophoresis (DEP) is a particle manipulation technique defined as particles movement in medium in presence of electrical fields (E-field). Particles that suspended in a medium can be manipulated and polarized by non-uniform E-field. The particle – medium interface will attract and accumulate all the charges inside the particles and the medium to their interface edges. An electric double layer will be formed by the accumulated electric charges. The relative quantity of these accumulated charges on the edges of the particle and the medium will be determined by the polarization of both medium and particle. Under E-field, positive and negative charges attract and bind to each other, and thus a dipole will be created. And the relative difference of positive and negative charges will determine the orientation of the dipoles created^32^. The net dipoles from the E-field results in a force that attract the opposite charges and repulse the identical charges. In the case of the uniform E-field when that generated by same size and shape of the electrodes with AC/DC source applied, particles under that E-field will be stationary because forces exerted on the particles from positive and negative charge will cancel each other, so the net force will be zero. However, the E-field will be non-uniform and inhomogeneous when created by different size, shape or non-parallel electrodes with AC/DC source applied. The net force on the particles in that field will not be zero. The movement of the spherical particle is the result of DEP force, and the force can be expressed as^33^:

$${F}_{DEP}=2\pi {\varepsilon }_{m}{r}^{3}Re({F}_{CM})\nabla {E}_{rms}^{2}$$

where ε_m_ is the permittivity of the medium, r is the radius of the particle, $\nabla {E}_{rms}^{2}$ is the gradient of the external E-field. *F*_*CM*_ is the Clausius-Mossotti factor, which has information about the dielectric properties of the particle and the medium, also the frequency dependence of DEP force. *F*_*CM*_ is given by^34^:

$${F}_{CM}=\frac{{\varepsilon }_{p}^{\ast }-{\varepsilon }_{m}^{\ast }}{{\varepsilon }_{p}^{\ast }+2{\varepsilon }_{m}^{\ast }}$$

ε_p_^*^ and ε_m_^*^ are the complex permittivity of the particle and medium, respectively. From the equation above, complex permittivity of the particle and medium are frequency dependent. The relation of ${{\rm{\varepsilon }}}_{p}^{\ast }$ and ${{\rm{\varepsilon }}}_{m}^{\ast }$ can be changed by adjusting frequency of the E-field. When ${{\rm{\varepsilon }}}_{p}^{\ast } > {{\rm{\varepsilon }}}_{m}^{\ast }$, the particle will be pulled to the region where the E-field is stronger, which is observed as positive DEP (pDEP) force. When ${{\rm{\varepsilon }}}_{p}^{\ast } < {{\rm{\varepsilon }}}_{m}^{\ast }$, the particle will be pushed away to weaker E-field region, which is observed as negative DEP (nDEP) force. DEP force will accelerate the particle inside the medium, and the acceleration is contributed by DEP force and the drag force. The drag force increases with the velocity of the particle moving inside the medium. When the DEP force equals to drag force, the particle reaches its equilibrium force. Usually, the particle reaches to its terminal velocity inside the medium after only a very short time^35^.

## Results

### Biosensor design

**Figure 1:**
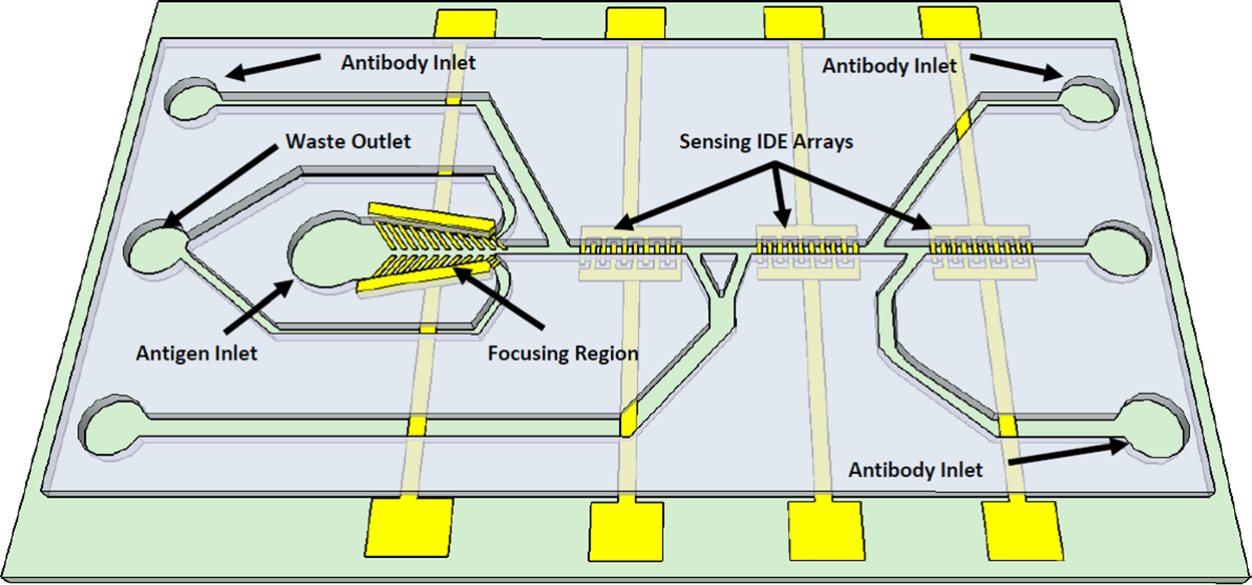
3D schematic of the impedance biosensor showing the focusing IDE array and three sets of the IDE arrays for three sensing regions. Each of the region could be used for sensing different serotypes of *Salmonella* without causing any cross-contamination.

The biosensor consists of two regions fabricated in SU8 microchannels as shown in Fig. 1. The focusing region is used for concentrating the *Salmonella* sample by getting rid of over 67% volume of the testing material. This has improved the device’s ability to detect bacteria at a low concentration. The focusing has the length of 3 mm and it consists of a vertical electrode pair along with 45° tilted thin film finger pairs with a ramp down channel with a start and end width of from 300 µm, and 75 µm, respectively. This electrode generates p-DEP forces that pushes the *Salmonella* cells toward the centerline of the channel and direct them toward the sensing microchannel. This has resulted in a concentrated sample. The bulk fluid will flow toward the outer channel into the waste outlets. The sensing region consists of three set of interdigitated electrode (IDE) arrays fabricated in the same microchannel that incorporate impedance measurement principles to detect the presence/ absence of *Salmonella*. Each electrode array has 20 finger pairs with finger length, width, and spacing between the fingers of 30 µm, 10 µm, and 10 µm, respectively. The bonding pads are stretched away to the sides of the device and used for impedance measurements. The sensing microchannel has a width and height of 25 µm, 25 µm, respectively. The three sensing electrodes were pre-functionalized by delivery specific anti-*Salmonella* antibody serotypes B, D and E, each for one electrode through independent antibody inlets without causing cross-contamination. All the antibodies were pre-linked with crosslinker. After the channel is filled with antibody solutions, the flow was stopped for 1 hour to ensure efficient adhesion of the antibodies to IDE arrays. The *Salmonella* samples were tested by flowing them through the sample inlet toward the focusing microchannel and continue flowing toward the sensing IDE arrays. After the sensing channel was filled with the testing sample, the flow was stopped for 30 minutes to facilitate the contact and binding between *Salmonella* antigens and the corresponding *Salmonella* antibody. It is noted that each device was used for one test to eliminate the possibility of sample contamination.

### Focusing demonstration using microbeads

**Figure 2:**
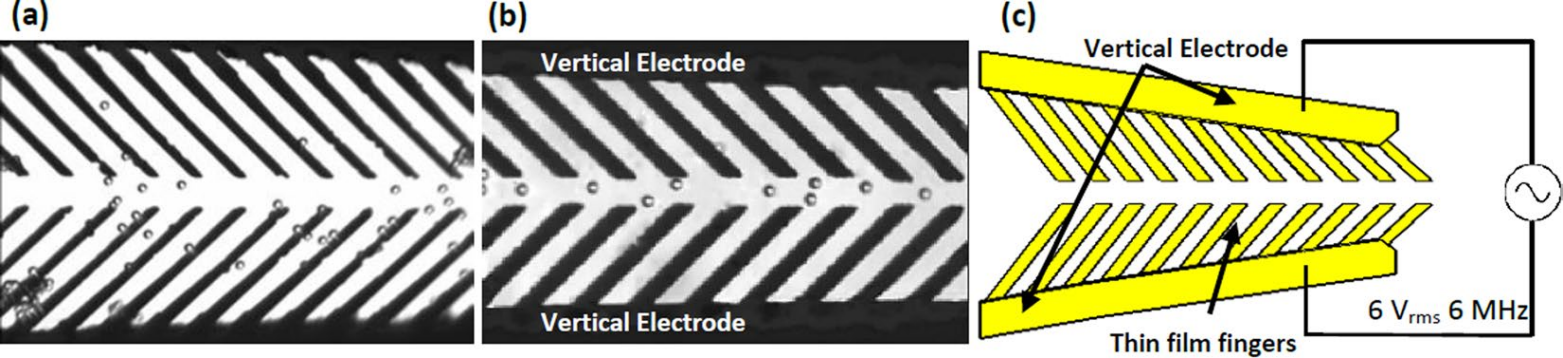
Optical Image of 4 µm microbeads (**a**) microbeads were dispersive at the beginning position of focusing region. (**b**) microbeads were concentrated to center of the channel at then middle position of the focusing region when focusing function is on. (**c**) schematics of the focusing electrode along with the applied voltage and frequency.

We first used polystyrene microbeads to test the focusing capability of the device. The microbeads were introduced into the channel via the sample inlet. When a AC signal (6 V peak-to-peak at 6 MHz from an AC power supply) was applied across the bonding pad, the focusing electrodes generates a non-uniform E-field and p-DEP forces the microbeads to move toward the center of the channel toward the region of high E-field. Figure 2 shows microbeads position before and after focusing turning on.

### Dose response

**Figure 3:**
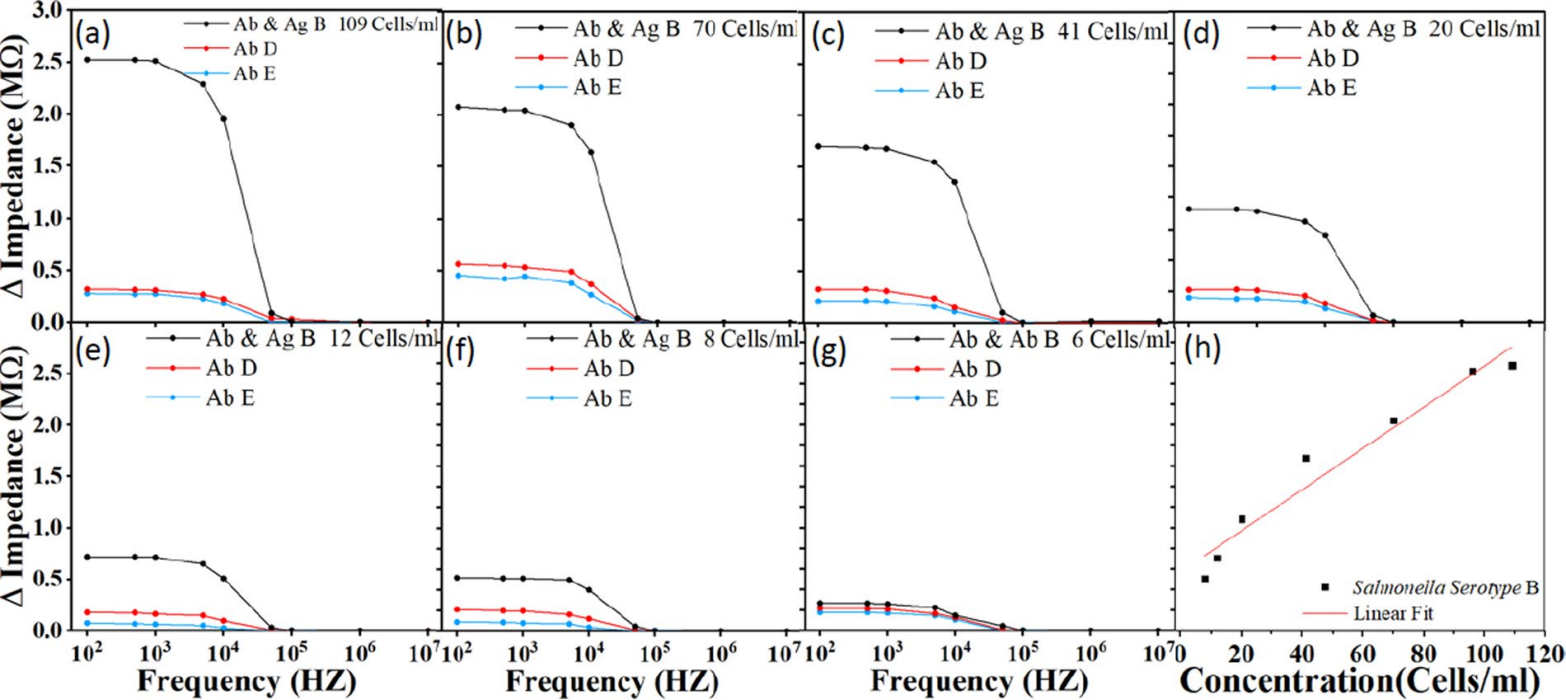
Impedance response for different concentrations of *Salmonella*. The sensor was first functionalized with crosslinked *Salmonella* antibody type B, D and E. *Salmonella* serotype B with concentrations of (**a**) 109, (**b**) 70, (**c**) 41, (**d**) 20, (**e**) 12, (**f**) 8 Cells/ml, spiked with ready-to-eat turkey sample was then delivered to the sensor for detection. The limit of detection was found to be 8 Cell/ml. (**g**) shows the variation of impedance change is directly proportional (linear) to bacterial cell concentration. The linear correlation coefficient is 0.955 and the sensitivity (Slope) is 0.02 MΩ per bacteria concentration decade. Ab stands for antibody, Ag stands for antigen.

The variations of the amount of substance on the surface of the electrodes cause the impedance to change. Impedance increases after antibody is immobilized on the IDE arrays compared to the IDE array immersed with just the antibody. After the *Salmonella* were selectively bound to the *Salmonella* antibody, the impedance was changed. The variation in impedance response is based on the concentration of the *Salmonella* as shown in Fig. 3, the sensor was tested with antibody serotypes B, D and E for each of the concentration. The impedance increases with higher concentration. The detection limit of the sensor was found to be 8 Cells/ml. For all concentrations, impedance decreases as function of frequency. All the sample concentration is pre-confirmed by culture method, so the number of bacteria colony is known before being delivered to the sensor for testing. The device cannot quantify the number of detected bacterial cells but it informs the presence or absence of bacterial cells. Experimentally, the impedance across the IDE array were measured and recorded after immobilizing the antibodies, and after the exposure of the target molecules (e.g., *Salmonella B*). Then, the antibody impedance was subtracted from the total impedance (after the exposure of the target molecules) and plotted the impedance difference, i.e., ΔImpedance (MΩ). To demonstrate the limit of detection, the device was tested using *Salmonella* B with a concentration of 6 Cells/ml. The impedance value is close to that of the antibody B, D and E. For example, the impedance reference reading after antibody coating was 5.22 MΩ, while the impedance after exposure to *Salmonella* B was 5.48 MΩ. The difference, 0.26 MΩ, is very small and hard to distinguish. Thus, 8 Cells/ml is the limit of detection. The *Salmonella* concentration was plotted as a function of impedance change from 8 Cells/ml to 109 Cells/ml in Fig. 3(h). The figure demonstrates that the variation of impedance change is directly proportional to bacterial cell concentration. In addition, the figure shows a linear correlation, ΔImpedance = Slope ∗ Concentration + Δo, where Δo is the intersection of the linear fit and the Y-axis. The correlation coefficient is 0.955 and the sensitivity (Slope) is 0.02 MΩ per bacteria concentration decade. The limit of detection is 8 Cells/ml.

### Simultaneous detection

**Figure 4:**
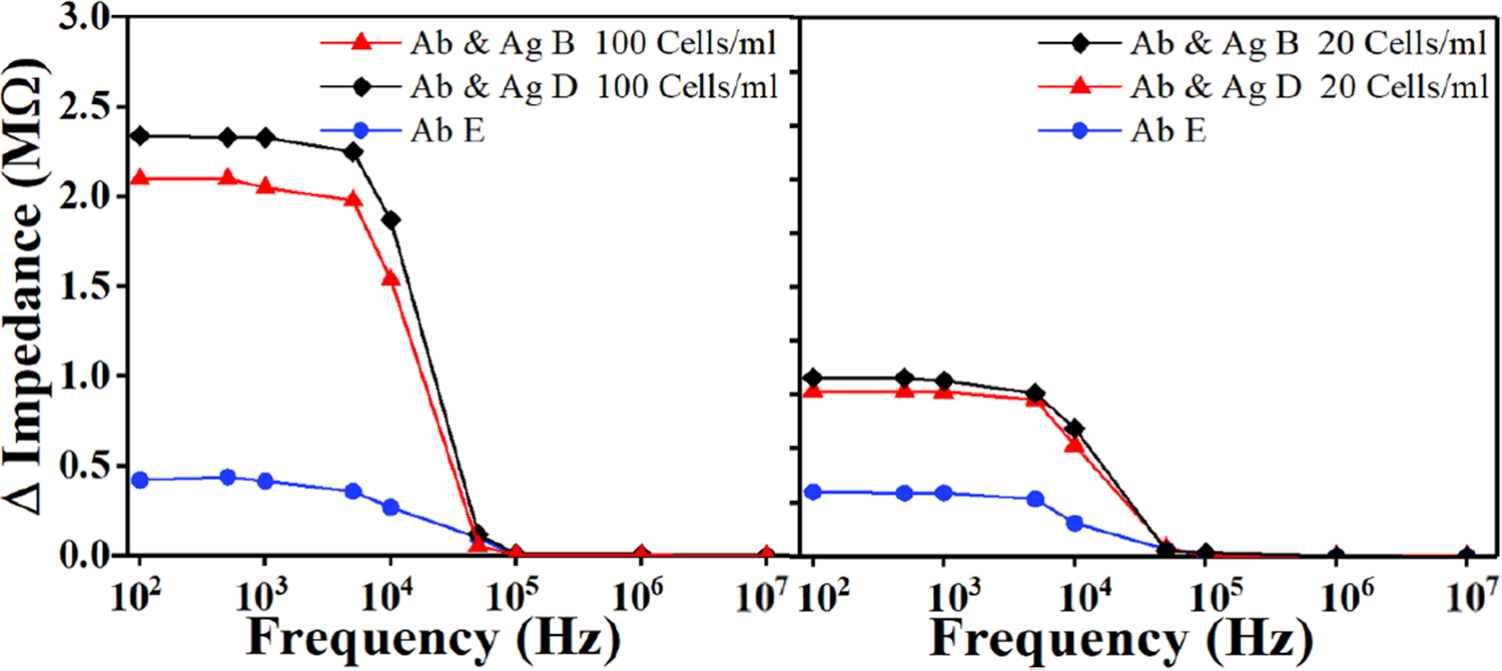
Sensor’s ability to simultaneously detect *Salmonella* serotypes B and D were tested by flowing two equal quantities mixture of the two different *Salmonella* serogroups for (**a**) 100 Cells/ml, and (**b**) 20 Cells/ml concentration. The figure shows the impedance change response as a function of frequency. The impedance of *Salmonella* antibodies B and D were subtracted from the measured impedance after the exposure of the target molecules and plotted the impedance difference. Ab stands for antibody, Ag stands for antigen.

Initially, the three mixtures of antibodies (B, D and E) were immobilized on the detection electrodes independently without causing any cross contamination such that *Salmonella* type B was placed on the first sensing electrode, D on the second sensing electrode, and E on the third sensing electrode. The impedance of each sensing electrode was recorded. After the *Salmonella* were injected to the device, the impedance was measured again. The experiments were performed using RTE turkey spiked with *Salmonella* type B and D with several concentrations. The impedance differences between antibodies and antigens were then calculated for each sensing region. The impedance change that demonstrates the device ability to simultaneously detect two *Salmonella* serotype B and D were plotted in Fig. 4. The results demonstrated a high impedance change using the electrode with *Salmonella* antibody B and D, while the other one electrode with antibody E had a weak signal.

### *Salmonella* detection with and without focusing function

**Figure 5:**
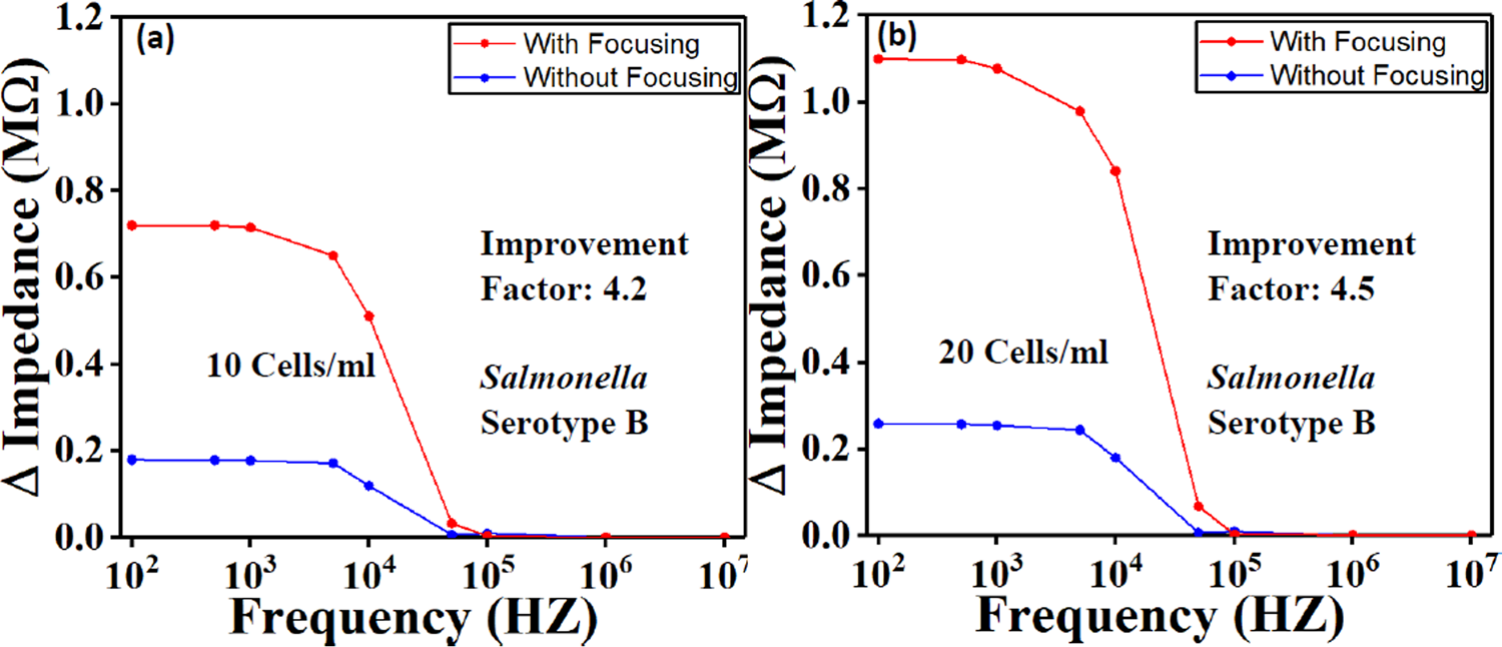
The comparison with and without focusing function on *Salmonella* with 2 concentrations (**a**) 10 Cells/ml and (**b**) 20 Cells/ml. The result has showed that the focusing function has improved the signal response by a factor up to 4.5.

Similar electrical field (amplitude and frequency) was applied to the biological sample to concentrate cells towards the sensing region. Experiments were conducted on *Salmonella* detection with and without the use of the focusing effect. The results demonstrated that the signal was improved by a factor range between 4–4.5 (Fig. 5).

### Selectivity on *Salmonella* and *E. coli*

**Figure 6:**
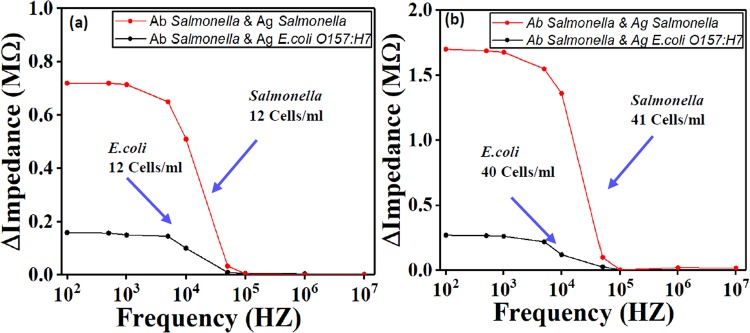
Test of sensor’s specificity to *Salmonella* serotype *B* for two concentrations (**a**) around 12 Cells/ml and (**b**) around 40 Cells/ml. The result show that *E. coli O157:H7* wound not bind to *Salmonella* antibody due to specificity. Ab stands for antibody, Ag stands for antigen.

In order to test the sensor’s selectivity on different types of antigen, *Salmonella* and *E. coli O157:H7* were used. The electrodes were coated with *Salmonella* antibody type B. the tested samples were spiked with *Salmonella* type B and *E. coli*. They were then introduced into the device separately in two different experiments. The result in Fig. 6 demonstrated that the device with *E. coli* antigens had a weak signal response while the other device with *Salmonella* type B antigens has a high signal response.

### Live and dead *Salmonella* cells differentiation

**Figure 7:**
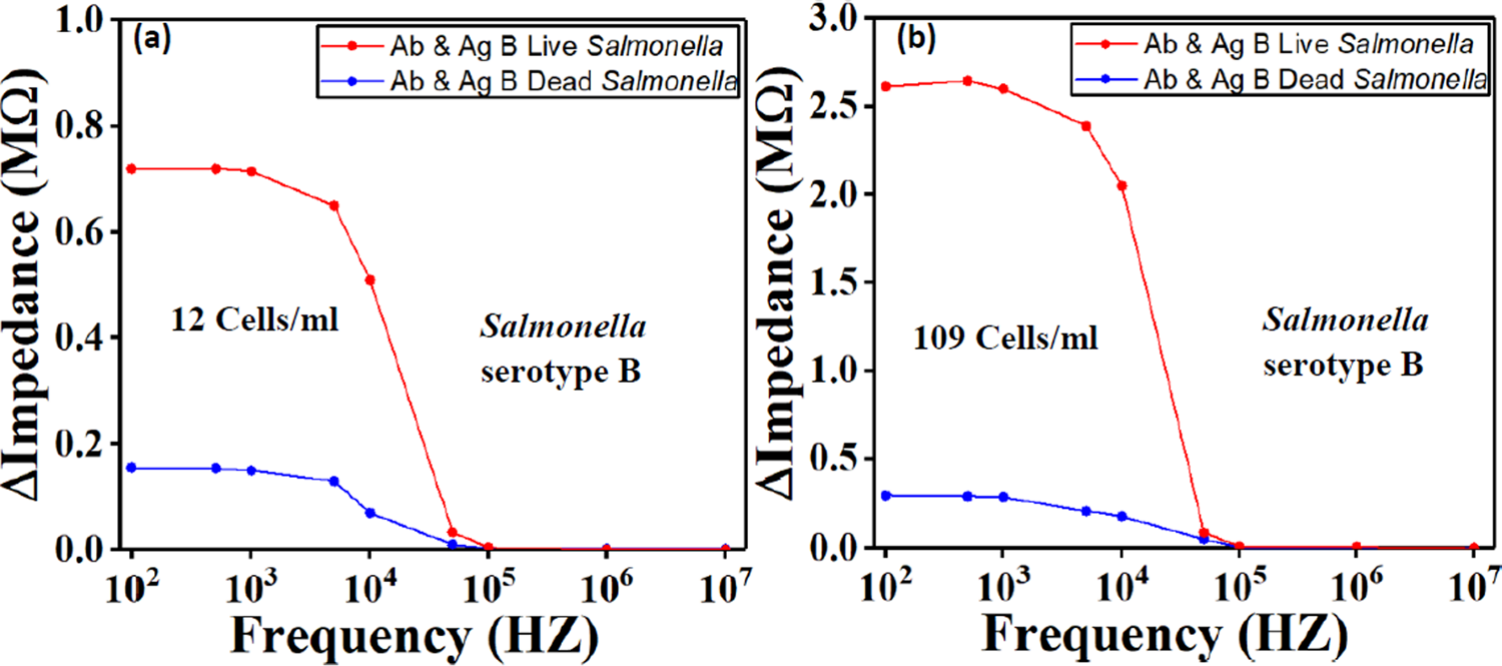
Comparison of live and dead *Salmonella* serotype B for two concentrations (**a**) 12 Cells/ml and (**b**) 109 Cells/ml. The results show the sensor’s capability to differentiate between live and dead *Salmonella*. Ab stands for antibody, Ag stands for antigen.

The biosensor was tested with live and dead *Salmonella* cells to demonstrate its ability to differentiate between them. The *Salmonella* bacteria were killed by brief exposure to high temperature which resembles real-life event. Two devices were coated with the anti-*Salmonella* serotype B, while dead and live bacteria were pumped into the two devices separately, each into one device. Figure 7 indicates that the impedance value obtained from the dead bacteria was close to baseline impedance, which was expected because the membrane of *Salmonella* antigen was damaged and thus lost the function to bonding with *Salmonella* antibody.

## Discussion

This paper presents design and fabrication of an impedance-based MEMS biosensor for different *Salmonella* strains detection. Rapid and quantitative detection of *Salmonella* in food source has been achieved. The design has single channel with focusing electrode and three sensing electrodes that make the sensor suitable for detections of three *Salmonella* serotypes simultaneously. The results demonstrate that the impedance increases when concentration of target bacteria increases, with the limit of detection found to be as low as 8 Cells/ml. Microbeads were used as an alternative for *Salmonella* cells in order to demonstrate the device focusing capability. The optimum parameters including frequency and AC voltage (V_p-p_) obtained with microbeads, were used for focusing *Salmonella*. The relative permittivity of the microbeads, and *Salmonella* are 4^36^, and 4.5–6.5^37,38^, respectively. The small difference may affect the optimum value of the operation frequency. However, it is noted that the frequency of operation is the dominant parameter that affect the focusing of microbeads and cells, especially the solution is the same for both experiments. By changing the frequency, we can switch between n-DEP, and p-DEP. The testing results demonstrated that the addition of focusing region have improved the response sensitivity by a factor between 4–4.5, while the total detection time was 45 minutes. The sensor can also differentiate dead from live cells and has a good selectivity on different *Salmonella* serotypes.

The results demonstrate the device advantages in terms of detection limit, required detection time and ease of operation. The impedance-based sensor in this study is suitable for detecting multiple pathogens simultaneously, which hasn’t been achieved by any other research groups. The ability to detect very low concentration (8 Cells/ml) in 45 minutes has granted the sensor a great potential to be used in the food industry. The current detection techniques which include bacterial culture, PCR, and ELISA do not allow timely assessment of safety of food products^39,40^ because they require 2–5 days^9,14^, and 1–2 days^41,42^, and 2–3 days^5–7^, respectively. In addition, PCR cannot distinguish between live and dead bacteria and false positives may occur^43,44^. Multiple biosensing techniques demonstrated high sensitivity including electrochemical biosensors, optical biosensors, and mass-based biosensors. However, none of these techniques met the USDA/FDA requirements. In addition, those devices can only do detection of one type of pathogens at a time.

The USDA/FDA set a zero-tolerance requirement for RTE poultry products. The testing method must meet the AOAC standard for certification, i.e.,1 cell/325 gr of product. Therefore, use of our device for RTE poultry products will require a short enrichment step. Due to the high sensitivity of our existing device, 8 Cells/ml, the length of the enrichment culture will be much shorter than with other technologies, such as ELISA and PCR. The duration of the enrichment step with our device is expect it to be around 6 hrs. This means the total detection time for our device will be approximately 7 hrs, which would mean that the results could be available within a typical production plant shift. In experiment, approximately 32.5 *Salmonella* cells were inoculated on 325 gr RTE Turkey and enriched in 2,925 ml BPW at 1:10 dilution and at 37 °C. After 4 hrs, the *Salmonella* concentration reached 7 Cells/ml. In addition, by applying the new USDA’s Laboratory Guidebook which suggests that the enrichment culture is done by inoculating 325 gr RTE poultry into 975 ml BPW at 1:4 dilution, a less time will be needed to reach the same pathogen concentration. For example, when the same number of *Salmonella* cells (32.5 cells) inoculated on 325 gr RTE Turkey sample, it took only 2 hours to reach 8 Cells/ml, which can be detected with our biosensing device. The low limit of detection (8 Cells/ml) indicates that our device will be able to approach the 1 cell/325 gr requirement within a production plant shift.

## Methods

### Biosensor fabrication

**Figure 8:**
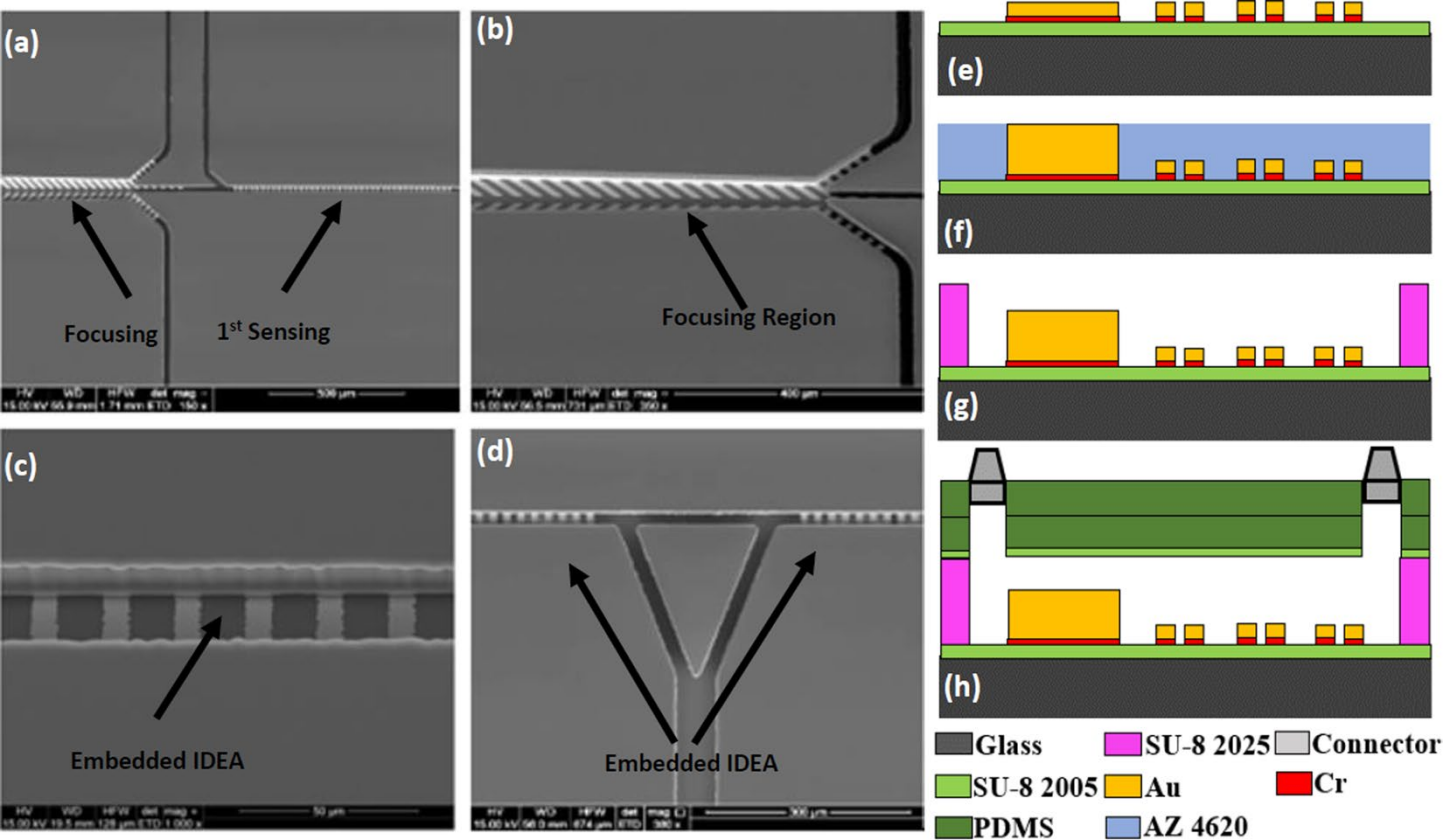
SEM of the sensor: (**a**) Focusing and 1^st^ Sensing region, (**b**) Magnified view of the focusing region. The excess waste will leave through the channels on two sides while the bacteria stay in the center and enter the sensing region, (**c**) Magnified view of the IDE arrays for sensing region embedded in the microchannel, (**d**) IDE arrays for 2^nd^ and 3^rd^ Sensing regions. The triangle part is used to split the fluid during antibodies collection for 2^nd^ and 3^rd^ sensing regions to reduce cross-contamination. Cross-sectional view demonstrating multiple layers of the sensor: (**e**) Electrode traces on top of the SU8 and glass slide, (**f**) Electroplating to increase the height of the focusing wall, (**g**) SU8 microchannel, (**h**) PDMS and fluidic connectors as seal.

**Figure 9:**
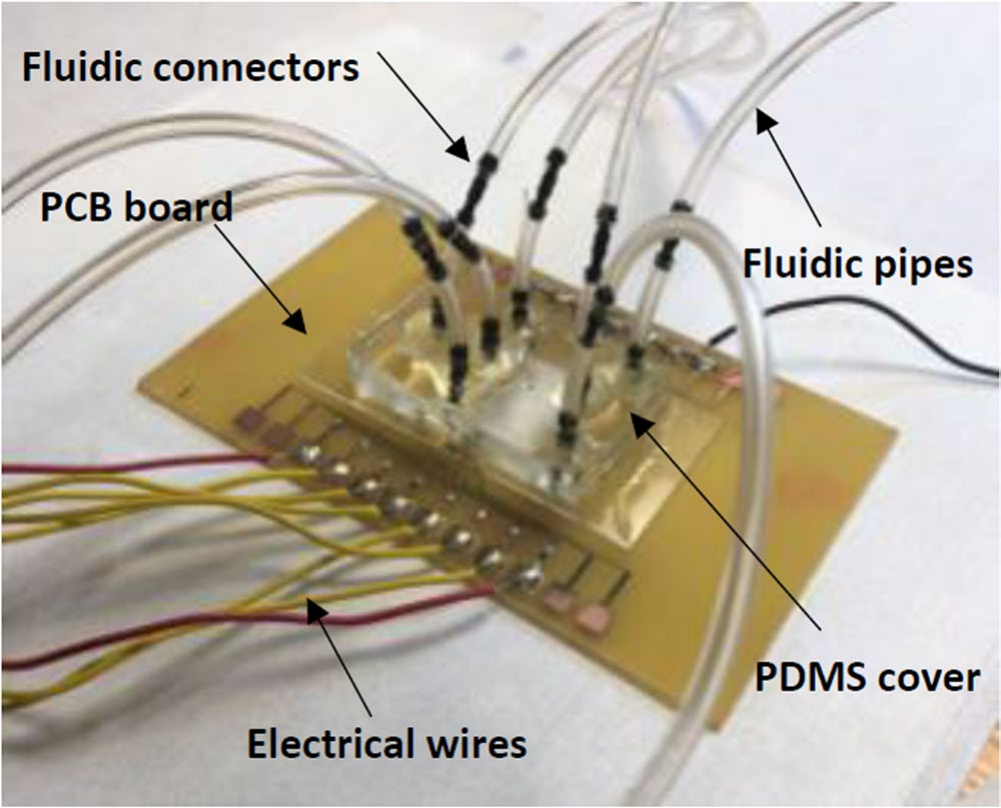
Completely fabricated sensor on a PCB board showing the fluidic connectors, tubes and wires.

The biosensor was fabricated on a 2 × 1.5 inch^2^ glass substrate using surface micromachining in following steps: (1) The glass slide was first cleaned using Piranha solution (hydrogen peroxide and sulfuric acid with a ratio of 1:3) for 4 minutes. (2) A layer of SU-8 (Microchem) with a thickness of 4 μm was spin coated on the surface of the glass slide, prebaked, exposed to UV light and hard bake at 150 °C for 30 minutes. (3) Chromium (Cr) and gold (Au) thin layers were deposited using DC sputtering with thickness of 50 nm and 150 nm respectively. (4) The Au thin film was patterned to form the IDE arrays, bonding pads, focusing fingers and seed layer for electroplating the focusing side walls. (5) Positive photoresist (AZ4620) with 15 µm thickness was patterned as a mold for electroplated Au to create the sidewalls. The Au was then electroplated for 15 μm. This is followed by Cr layer etching. (6) The microchannel was formed using SU-8 2025 negative photoresist with a thickness of 25 μm. (7) Two thick layers of polydimethylsiloxane (PDMS) were prepared and cured. The first one is used to cover the microchannel, while the second layer serves as top cover with fluidic connectors. Oxygen plasma treatment was used on the first layer to change its surface to be more hydrophilic before it was aligned and bonded to the glass substrate. Then it was baked on a hotplate at 65 °C for 5 minutes to ensure the bonding between the PDMS and the glass substrate is secured. The second PDMS slab with inlet and outlet fluidic connectors were then bonded to the first layer of PDMS using oxygen plasma. Epoxy glue was used to seal the fluidic connectors further to improve the device reliability. Scanning electron micrographs (SEMs) of the fabricated devices are shown in Fig. 8(a,d). Figure 8(e,h) is the fabrication of the sensor step by step. A completely packaged sensor in PCB board in Fig. 9 has shown the fluidic connectors, tubes and electrical wires.

### Microbeads for focusing function demonstration

The 4 μm diameter DUKE STANDARDS^TM^ Microbead with 1% coefficient of variation was purchased directly from ThermoScientific. The microbeads were made from polystyrene and suspended in water-based solution before use. The relative permittivity of the microbeads to free space is 3.8 (the permittivity of free space is 8.854187817 × 10^−12^ C^2^/(Nm^2^)).

### Culture and cell preparation *Salmonella* culture preparation

Ready-to-eat turkey breast (RTE) turkey breast was purchased from a grocery store and stored at 4 °C until use. Each 325 g RTE turkey breast was weighted and placed in a sterile bag, 2925 ml buffered peptone water was poured into the bag, and the bag was shaken for one minute. The supernatant was then filtered through a 100 μm and then a 20 μm cell strainer (pluriSelect Life Science, Leipzig, Germany) to remove big debris which may block the biosensor microchannels. The filtered rinse was used freshly to dilute *Salmonella* cells or aliquoted and frozen at −20 °C until used.

### *Salmonella typhimurium* preparation

An avirulent *Salmonella* enterica Typhimurium strain (ΔsipB, Cmr) was used to spike RTE turkey breast. An overnight culture (37 °C, 200 rpm, in LB broth) of S. enterica Typhimurium was harvested by centrifugation at 4000 rpm 10 minutes and washing with sterile distilled water 3 times and then suspended in 25% sterile glycerol. The cell suspension was aliquoted and frozen at −80 °C until used. At the same time, one aliquot was serially diluted and plated on LB agar plates to determine the cell concentration. The cell concentration was determined to be 2 × 10^9^ Cells/ml. Before the test, one aliquot *Salmonella* suspension and several aliquots of RTE turkey rinse were thawed on ice. The *Salmonella* suspension was then diluted with the of filtered RTE turkey rinse to desired concentrations.

### Antibody preparation

Rabbit anti-*Salmonella* O antiserum poly B, D, and E (Becton, Dickinson and Company, Franklin Lakes, NJ) were used as capture antibodies. The crosslinker, sulfosuccinimidyl 6-[3-(2-pyridyldithio) propionamido] hexanoate (sulfo-LC-SPDP) (Fisher Scientific, Hampton, NH), was used for antibody immobilization. Briefly, for each test, 8 μl of each antiserum was diluted with 292 μl filtered chicken rinse, mixed with 300 µl sulfo-SPDP (20 mM water solution), and then incubated at room temperature for 1 hour. To reduce the disulfide bond of the thiolated antibody, 200 μl DTT (0.1 M sodium acetate buffer, 0.1 M NaCl, pH 4.5) (Fisher Scientific, Hampton, NH) was then added into the tube to react for 30 min at room temperature before the antiserum mixture was loaded into the biosensor. The 1:100 diluted antiserum-crosslinker mixtures were delivered into the device using three syringe pumps through the antibodies’ inlets, respectively. The electrode surfaces were then functionalized with the three antiserum-crosslinker mixtures, one for each channel and without causing any cross contamination. The flow was stopped to all the three channels and allowed 1 hour for the antibodies to get immobilized on the gold electrodes to achieve good bonding to the electrodes thus providing high specificity. A washing step was performed after 1 hour to remove any unbounded antibodies by pumping distilled water inside the three channels.

### Antibody crosslinker

A new immobilization method using cross-linker was used to exploit natural and direct covalent bond formation between gold electrode and a thiolated antibody. The cross-linker we used is Sulfo-LC-SPDP, it is a thiolation reagent that introduce available sulfhydryl groups to an antibody. A monolayer coating of the thiolated antibody onto the gold surface can be easily achieved because the reaction potential between the gold and sulfur is very strong, which achieved 50% in signal response^45,46^. In our experiments, all the antibodies were prepared with crosslinker.

### Antibody immobilization

The mixtures of antibody with cross-linker were delivered to the device through inlets using syringe pumps at a constant flow rate. Subsequently, the electrode surface was then functionalized with the antibodies, one for each sensing region without causing any cross contamination. The flow was stopped for 1 hour to allow the antibodies to get immobilized on the gold electrodes firmly to achieve a good and specific bonding between the antibodies and the gold electrodes. A washing step by pumping DI water into the channel is then performed after 1 hour of the immobilization to remove excess antibodies and other waste materials. The flow for all fluids was set to be 2 µL/min to prevent fluids from flowing in random fashion.

### Antigen capturing

Ready to eat turkey (RTE) breast meat was stored at 3 °C before use. An avirlent *S*. *enterica* Typhimurium strain (*ΔsipB, cat*+) (serotype B) was used for spiking turkey meat. The RTE samples spiked with *Salmonella* strain were then introduced into the channel through the sample inlet by a syringe pump at a constant flow rate. After the channel was filled with sample, the flow was stopped for 30 minutes to allow the biological interaction between the antibodies and the antigens to form antibodies-antigens binding. The same washing step and flow rate were used again to remove unbounded cells and excess reactants. Impedance was then measured by Agilent 4294A impedance analyzer. The device will be discarded after being used for one time.

## Data Availability

The datasets generated during and/or analyzed during the current study are available from the corresponding author on reasonable request.
